# Impact of a combined multimodal-aerobic and multimodal intervention compared to standard aerobic treatment in breast cancer survivors with chronic cancer-related fatigue - results of a three-armed pragmatic trial in a comprehensive cohort design

**DOI:** 10.1186/s12885-017-3142-7

**Published:** 2017-03-02

**Authors:** Matthias Kröz, Marcus Reif, Augustina Glinz, Bettina Berger, Andreas Nikolaou, Roland Zerm, Benno Brinkhaus, Matthias Girke, Arndt Büssing, Christoph Gutenbrunner, On behalf of the CRF-2 study group, Danilo Pranga, Fadime ten Brink, Anette Zander, Annette Weninger, Nina Klara, Mahmud Naghavi, Peter Klug, Andrea Küssmann, Petra Wundram, Michaele Quetz, Sarah Zastrutzki, Dorothea Friemel, Anne Kristin Meyer, Judith Schmotz, Martina Jackmuth, Suzanne Mehrtens, Sarah Zastrutzki, Christian Heckmann, Barbara Trapp, Ursula Heusser, Frederike Rettigand, Elisabeth Rieger, Astrid Didwiszus, Béatrice Gelin-Kröz, Karen Baumhöver-Wegener, Birgit Lindemann, Sonja Steffens, Ramona Beutke

**Affiliations:** 1 Department of Internal Medicine, Havelhöhe Hospital, Kladower Damm 221, D-14089 Berlin, Germany; 2 Research Institute Havelhöhe, Kladower Damm 221, Berlin, D-14089 Germany; 3 Institute for Social Medicine, Epidemiology and Health Economics, Charité Universitätsmedizin Berlin, Charité CCM, Berlin, 10098 Berlin Germany; 4 Institute for Integrative Medicine, University of Witten/Herdecke, Gerhard-Kienle-Weg 4, Herdecke, 58313 Germany; 5 Society for Clinical Research, Hardenbergstraße 20, Berlin, 10623 Germany; 6 Clinic for Rehabilitative Medicine, Hannover Medical School, Carl-Neuberg-Straße 1, Hannover, 30625 Germany

**Keywords:** Aerobic training, Anthroposophic medicine, Cognitive behavior therapy, Breast cancer, Cancer-related fatigue, Eurythmy therapy, Multimodal intervention, Painting therapy, Sleep education, Sleep restriction

## Abstract

**Background:**

Cancer-related fatigue (CRF) and insomnia are major complaints in breast cancer survivors (BC). Aerobic training (AT), the standard therapy for CRF in BC, shows only minor to moderate treatment effects. Other evidence-based treatments include cognitive behavioral therapy, e.g., sleep education/restriction (SE) and mindfulness-based therapies. We investigated the effectiveness of a 10-week multimodal program (MT) consisting of SE, psycho-education, eurythmy- and painting-therapy, administered separately or in combination with AT (CT) and compared both arms to AT alone.

**Methods:**

In a pragmatic comprehensive cohort study BC with chronic CRF were allocated randomly or by patient preference to (a) MT, (b) CT (MT + AT) or (c) AT alone. Primary endpoint was a composite score of the Pittsburgh Sleep Quality Index and the Cancer Fatigue Scale after 10 weeks of intervention (T1); a second endpoint was a follow-up assessment 6 months later (T2). The primary hypothesis stated superiority of CT and non-inferiority of MT vs. AT at T1. A closed testing procedure preserved the global α-level. The intention-to-treat analysis included propensity scores for the mode of allocation and for the preferred treatment, respectively.

**Results:**

Altogether 126 BC were recruited: 65 were randomized and 61 allocated by preference; 105 started the intervention. Socio-demographic parameters were generally balanced at baseline. Non-inferiority of MT to AT at T1 was confirmed (*p* < 0.05), yet the confirmative analysis stopped as it was not possible to confirm superiority of CT vs. AT (*p* = 0.119). In consecutive exploratory analyses MT and CT were superior to AT at T1 and T2 (MT) or T2 alone (CT), respectively.

**Conclusions:**

The multimodal CRF-therapy was found to be confirmatively non-inferior to standard therapy and even yielded exploratively sustained superiority. A randomized controlled trial including a larger sample size and a longer follow-up to evaluate multimodal CRF-therapy is highly warranted.

**Trial register:**

DRKS-ID: DRKS00003736. Recruitment period June 2011 to March 2013. Date of registering 19 June 2012.

## Background

Cancer-related fatigue (CRF) is a major issue in cancer patients. In a British study 58% of all cancer outpatients report fatigue to affect them ‘somewhat to very much’, therewith denoting it as the most important yet insufficiently treated symptom burden [1]. CRF is defined as ”a distressing, persistent, subjective sense of physical, emotional and/or cognitive tiredness or exhaustion related to cancer or cancer treatment that is not proportional to recent activities and interferes with usual functioning…“ [2]. Apart from tumor disease and burden, CRF is mainly caused by chemo-, radio and possibly anti-hormonal treatment [2]. A new study reports high fatigue levels to be associated with gene polymorphism causing a high expression in promoter regions of three different interleukins (IL1B, IL6, TNF alpha) [3]. Sleep disorders, particularly insomnia, psychological distress or further chronic internal medical conditions constitute modulating and aggravating factors [4]. In 34% of breast cancer survivors CRF persists after 5 to 10 years [5], and global and cognitive fatigue are still captured after 10.6 years in 17 and 28% of patients, respectively [6]. In this population CRF is a frequent reason for invalidity pensions in Germany [7]. The treatment of CRF with the best available evidence in breast cancer is aerobic training, showing a minor to moderate (standardized mean-differences) effect size of 0.27 to 0.315 [8, 9]. Cognitive behavioral approaches such as psycho-education yield minor effect sizes [9], while sleep educational approaches including sleep-restriction and stimulus control [10, 11] show minor to moderate effect sizes comparable with mindfulness-based interventions [12, 13]. For pharmacological therapies the evidence is still unclear [14] even though some authors have recommended stimulants such as Ritalin or Modafinil in case of severe fatigue [15]. Another pharmacological approach is mistletoe treatment, for which positive results in the reduction of fatigue have been published; trials using sufficiently robust fatigue measures with CRF as primary outcome are still lacking [16].

Due to the difficulty in CRF treatment and the unsatisfactory results of mono-therapies a multidisciplinary therapy appears to be a more appropriate approach [17] with slightly better effect-sizes on fatigue for a combined aerobic training and myofascial release massage compared to the above cited meta-analysis for aerobic training alone (moderate effect-sizes = 0.52) and significantly better than usual care [18]. Therefore, we developed a 10-week intervention program based on Anthroposophic Medicine [19] including psycho-education, sleep-education, a mindfulness-oriented movement therapy (eurythmy therapy) and painting therapy which significantly improved CRF in a pilot study, and sleep quality and autonomic regulation [20]. In this paper we report on a trial comparing the multimodal (sleep-education, psycho-education, eurythmy, and painting therapy) therapy and a combination of multimodal and aerobic treatment with the standard therapy, i.e., aerobic training only.

## Methods

A prospective, parallel, open-label pragmatic trial was conducted in three centers, the 1) Research Institute Havelhöhe and Department of Internal Medicine, Hospital Havelhöhe, Berlin, 2) Center of Integrative Medicine, University Witten/Herdecke and Department of Gynecology, Hospital Witten/Herdecke, and 3) in the Department of Rehabilitation Medicine, Hannover Medical School, all located in Germany, from June 2011 to December 2013. The study had a comprehensive cohort design, i.e., patients could decide if the study intervention was chosen based on their own preference or by randomization.

The study was an investigator initiated trial, conducted according to the declaration of Helsinki, approved by the responsible local ethics committees and subject to GCP-conform on-site monitoring. The trial is registered in the German register of clinical studies (DRKS-ID: DRKS00003736).

### Patients

Most patients were recruited through local newspapers and through physicians who informed their patients of our study; other patients spontaneously contacted the study centers. All participants signed an informed consent form after the study had been explained to them.

Patients were eligible if they were female breast cancer patients between 18 and 75 years old, had a diagnosis of chronic Cancer-related Fatigue for more than 6 months (Fatigue Numerical Scale ≥ 4 and Cancer Fatigue Scale (CFS-D ≥ 24)), a history of at least 36 months since surgery/the end of adjuvant (radio-) chemotherapy and a maximum of 45 months after first diagnosis. Exclusion criteria are listed in Table 1.

**Table 1:** Exclusion criteria

• metastases,
• (radio-)chemotherapy or surgery in the last 6 months,
• anemia (hemoglobin <10 mg/dl),
• other severe chronic conditions:
- heart insufficiency > NYHA 1°,
- instable angina pectoris > NYHA 1,
- peripheral arterial occlusive disease > stage 1,
- COPD > stage 2,
- chronic renal insufficiency (creatinine > 2,5 mg/dl),
- manifest non-treated hypothyreosis (TSH > 4 mU/l, fT4 < 9 pmol/l) or hyperthyreosis (TSH <4 mU/l, fT4 > 24 pmol/l),
- severe limitations of musculoskeletal system,
- manifest major-depression or psychosis,
- sleep-disorders such as untreated sleep apnea syndrome,
- untreated relevant restless legs-syndrome or narcolepsy,
• ongoing erythropoietin-therapy or transfusions,
• intensive training with optimized physical training of more than 2 × 30 min per week,
• psychotherapy started within the last 3 months or specified CRF-education or sleep education within the last year.

### Treatment allocation and randomization

All patients received information about the study design and study interventions to support them in their decision to be allocated to one of the intervention groups based on their own preference or by randomization. Balanced randomization lists (i.e., group proportions of 1:1:1) with varying permutation block sizes were separately created for each center beforehand. The randomized allocation was conducted by a central randomization service via fax at the Institute for Clinical Research, Berlin. No concealment of treatments was carried out.

### Procedure and intervention

The study interventions, multimodal therapy (MT) and the multimodal-aerobic combination therapy (CT) were developed at the Research Institute Havelhöhe, Berlin, defined in an intervention manual resulting from a consensus process involving a group of experts (internists, oncologists, specialists in sleep medicine, psycho-oncologists, physio-, painting-, and eurythmy therapists) [21] and tested for feasibility in a pilot-study [20].

**Fig. 1:**
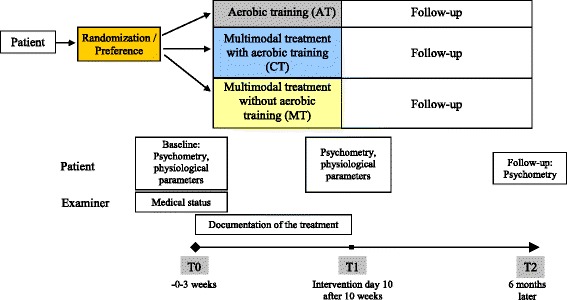
Overview of the study design

After capturing baseline measurement (T0) the outcome parameters were to be evaluated after an intervention period of 10 weeks (T1) and a subsequent follow up period of 6 months (T2) (Fig. 1).

MT was to be carried out with an intensity of 140–165 min per week and an additional 15 min for debriefing. According to protocol, the intervention was planned to take a total of 1450 min over 10 weeks. In CT the length of therapy was to be 165–185 min with an additional 15-min period for debriefing. The intervention took a total of 1810 min over a 10-week period.

### Psycho-education (MT/CT)

Following a feedback procedure at the beginning of each session the psycho-oncologist gave information on, and highlighted the understanding of illness, processing of illness and dealing with distressing feelings and thoughts, promotion of mental and physical health, social support and communication, personal responsibility, inner concentration exercises, stress management, and issues of reorientation [22]. After each session the participants were given exercises to be carried out until the next psycho-education session.

### Sleep education (MT/CT)

Patients attended an information session on the basics of chronobiology and sleep. This education aimed to contribute towards an improved understanding of sleep, and therefore also consecutively to an improvement in the ability to self-manage sleep problems and the stabilization of day and night sleep hygiene. Patients were also asked to fill out a sleep diary, once prior to the start of the intervention and a second time after two individual adjustments of the sleep rhythm in accordance to the sleep-restriction and stimulus protocol recommendations [23, 24].

### Eurythmy therapy (MT/CT)

Eurythmy therapy is a mindfulness-based movement therapy used in anthroposophic medicine for more than 90 years which expresses sounds and rhythms as movements and gestures [25]. The following exercises, such as I-A-O, clenching-spreading, striding, rhythms/hexameter, the vowel”Ei“, and consonants which should achieve a rhythmic stabilisation such as L, M, and R, were practised for the improvement of breast cancer-related dysrhythmia [26]. The so-called ”cancer series“ O-E-M-L-EI-B-D was also introduced and practised [20, 25]. Each session was followed by a rest period of 15 min.

### Painting therapy (MT/CT)

Painting therapy was implemented in Anthroposophic Medicine by Margaretha Hauschka [27]. Its aim is to stimulate the cognitive and affective functions [20]. Each therapy session started with a 10-min period where patients were asked to draw shapes. Then patients were asked to produce a series of paintings with a rhythmic development of day/night motifs and a light/darkness spectrum using water colors, starting with a painting in blue and the gradual reinforcement of yellow and brightness, followed by the integration of red [20].

### Aerobic training (AT/CT)

Aerobic training is the actual gold-standard treatment for CRF and was used in the control arm (AT) and in the multimodal-aerobic combination therapy (CT). The aim of this intervention was to achieve a 70–80% exposure in order to improve the endurance and physical performance and with this the global and physical fatigue [28]. At the beginning, patients’ performance status was individually evaluated by ergometry test with increasing steps by 25 watt every two minutes and heart rate being monitored at the end of each step [29]. In the course of the trainer-controlled therapy, performance adjustments were carried out on the basis of heart rate monitor watches [29]. Eight trainer-led 45-min sessions were carried out inclusive of a rest period, which were complemented by home-based training (3–5 × 30 – 45 min per week).

### Outcome measures

Primary endpoint was a composite outcome of the Pittsburgh Sleep Quality Index (PSQI) and the Cancer Fatigue Scale (CFS-D) after 10 weeks (T1); a second endpoint was assessed after a follow-up period of 6 months (T2).

The German version of CFS-D is a 15-item scale with three subscales (physical, cognitive and affective fatigue). The CFS-D consists of a five-point Likert scale (0–4), with its global scale ranging from 0 (no fatigue) to 60 (maximum fatigue). The CFS was developed in Japan [30], and validated in German as the CFS-D [31]. The CFS-D is highly reliable (Cronbach’s-alpha r_α_ = 0.94, test-retest reliability r_rt_ = 0.82) with a robust validity [31] and classifies values ≥ 30 as clear symptoms of fatigue, ≥ 24 points as suspected moderate fatigue and ≤ 23 as no or only minor symptoms.

The PSQI is a widely used questionnaire which captures sleep quality and attitudes. Along with qualitative items, the scale consists of 18 quantitative items split into seven components with values ranging from 0 to 3 (sleep quality, sleep latency, sleep duration, sleep efficiency, sleep disturbances, use of hypnotics, and daily sleepiness). The overall sum-scale detects the global sleep quality ranging from 0 (good sleep quality) to 21 (maximum sleep disturbances) [32].

### Safety

At each intervention, any subjective health complaints reported by patients were recorded independent of a possible relation to the study interventions and graded according to the Common Toxicity Criteria for Adverse Events. Incidences representing serious adverse events (SAEs) were to be notified within 24 h to the Safety Board.

### Statistics

#### Testing strategy

To avoid multiple testing inflation of the alpha error the analysis included two provisions, the combination of CFS-D and PSQI into an univariate composite score derived from their joint principal component (PC-score) [33], and the use of a closed testing procedure [34]. The comparison of the treatments started with a combined test for superiority of CT and for non-inferiority of MT compared to AT. Only if this overall test was statistically significant could the separate comparisons of CT and MT to AT be evaluated in a confirmative intention.

#### Sample-size estimation

For the comparison between CT and AT the smallest clinically relevant difference, i.e., 5% of the respective parameter range, was assumed for both outcome measures [35]. Standardized effect sizes (Cohen’s d) were calculated using standard deviations derived from the pilot study [19]. Sample size estimation was based on the combined test for superiority of CT and for non-inferiority of MT compared to AT. As non-inferiority thresholds (δ) a uniform standardized effect size of δ = 0.2 was chosen for both CFS-D and PSQI due to medical and statistical reasons. Since no sample size formula for Läuter’s PC-score exists, a marginal modeling approach for correlated parameters based on general estimation equations was used [36]. Further estimations were done by simulation using random datasets. The most conservative estimation yielded a total sample size of 114 patients.

#### Statistical analysis

According to the primary hypotheses of this study a) CT is superior to AT, and b) MT is at least as effective as AT in improving the composite endpoint of CFS-D and PSQI after 10 weeks of intervention. The general linear model used for testing both hypotheses included the PC-score at T1 as dependent variable, the baseline PC-score at T0 as covariate, and the treatment arm and preference/randomization status as independent factors. Since preference for a certain treatment may result in a biased outcome two propensity scores (PS) were additionally included in the model as continuous covariates aiming to account for a) treatment allocation by preference versus randomization, and b) preferring AT over MT, respectively. The logistic regression models for calculating the PS included age and vital signs, anamnestic and socio-demographic parameters, baseline values of the efficacy parameters, and questionnaires regarding patients’ expectations, autonomic and self-regulation [37]. Exploratory analyses assessed the PC-score at T2 as well as the CFS-D and PSQI and their respective subscales separately at T1 and T2. Changes from baseline are descriptively expressed as standardized effect sizes to enable a direct comparison of all parameters [38].

All analyses were based on the intention-to-treat principle, i.e., including all patients with valid screening/baseline measurements at T0. Missing questionnaire items were substituted according to their respective manuals. For the primary effectiveness analysis missing data at T1 were imputed by last-value-carried-forward (LOCF) as provided for in the protocol. Sensitivity analyses were done using worst-case and multiple imputations of missing data, omitting PS from the model, and using PS values derived from various models. PS never showed a significant influence, and even the restricted model without PS gave an equivalent outcome (results not shown).

Adverse events were analyzed with respect to the absolute number of affected patients and frequency of incidences. Only treatment-emergent signs and symptoms (TESS, i.e., between start of treatment and 4 weeks after last therapy visit) were further examined. Relative frequencies were calculated with reference to a single application and to the total length of patients’ therapy periods. Treatment groups were compared with regards to relative frequency and severity of TESS using a Poisson model accounting for differences in patients’ treatment durations.

All tests were carried out two-sided at an alpha error level of α = 5% and with corresponding 95% confidence intervals. Randomization lists, sample size estimates and statistical analyses were produced using SAS® version 9.1 (SAS® Institute, Cary, NC, USA).

## Results

**Fig. 2:**
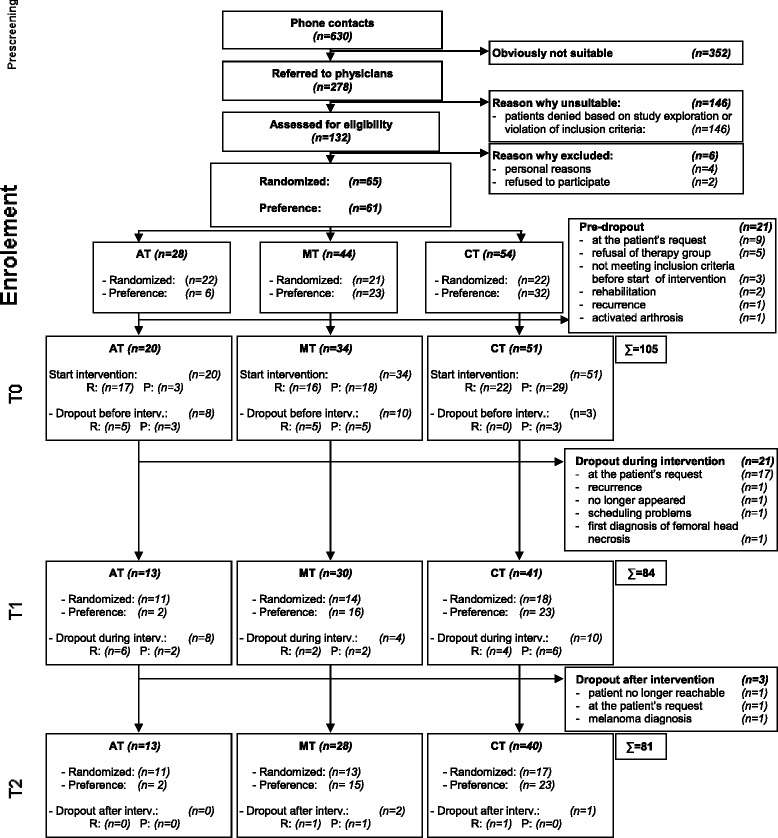
Flow chart for recruitment

Between June 2011 and March 2013 278 breast cancer patients were referred to the study physicians for screening. Of these, 132 breast cancer patients with a significant chronic cancer-related fatigue were assessed for eligibility and from these 126 were included in the study (65 randomized and 61 assigned by preference; Fig. 2). 28, 44 and 54 patients were randomly allocated to/ preferred the aerobic (AT: 22/ 6), multimodal (MT: 21/ 23) and combination arm (CT: 22/ 32), respectively. 20 patients who did not participate in the baseline assessment or any intervention were excluded from the Intention-to-Treat analysis (ITT). One AT patient had to be excluded before intervention due to an activated arthrosis. Of the remaining 105 patients included in the ITT analysis, 84 finished the intervention (13/ 30/ 41), and 81 patients (13/ 28/ 40) completed the 6-month (T2) follow-up. (Fig. 2: Flow chart).

### Study-group characteristics

Patients’ mean age ranged from 56.4 to 58.8 without showing significant differences between intervention groups. Similarly, the study arms were comparable with regard to time since first diagnosis, tumor biology, stage and treatment, or to socio-demographic characteristics (Table 2) except of a smaller average height in the AT-group (163 cm compared to 168 cm and 167 cm in the CT and MT group, respectively; *p* = 0.0168), less HADS-anxiety (MT: mean = 7.3 (SD = 3.1); AT: mean = 9.4 (4.3); CT: mean = 9.3 (3.5); *p* = 0.0263), less rehabilitation (MT = 11.76%; AT = 35%; CT = 31.37%; *p* = 0.027) and ‘other disorders’ (*p* = 0.0313) in the MT group compared to AT and CT. Nevertheless, baseline values of CFS-D and PSQI were comparable (Table 3). The factors most prominently contributing to the PS regarding randomization or preference included patients’ ‘expectations to better cope with daily life and deal with the disease’, actual hormonal therapy, concomitant medication, diastolic blood pressure, participation in rehabilitation programs, and the score of the questionnaire on self-regulation [39]. Factors mostly contributing to the PS regarding the preference for MT over AT included tumor stage, actual tumor aftercare, vocational training, and patients’ expectation of a better physical condition.

**Table 2:** Socio-demographic characteristics at baseline

Treatment group	Aerobic treatment	Multimodal treatment	Combination treatment	*p*-value
Included	28	44	54	
Started Intervention T0	20	34	51	
Completed T1	13	30	41	
Marital status				
Single (%)	1 (5.00)	7 (20.59)	8 (15.69)	0.5994
Married (%)	14 (70.00)	16 (47.06)	27 (52.94)	
Divorced (%)	3 (15.00)	8 (23.53)	13 (25.49)	
Widowed (%)	2 (10.00)	2 (5.88)	2 (3.92)	
Missing data (%)	0 (0.00)	1 (2.94)	1 (1.96)	
Children: yes (%)/ Children at home: yes (%)	16 (80.00)/ 6 (30.00)	23 (67.65)/ 8 (23.53)	38 (74.51)/ 11 (21.57)	0.3481/ 0.6599
Employment				
Employed (%)	9 (45.00)	10 (29.41)	25 (49.02)	0.1242
Housewife (%)	1 (5.00)	3 (8.82)	1 (1.96)	
Unemployed (%)	0 (0.00)	1 (2.94)	6 (11.76)	
Pensioner (%)	6 (30.00)	13 (38.24)	11 (21.57)	
Sickness certificate (%)	3 (15.00)	4 (11.76)	4 (7.84)	
Other (%)	0 (0.00)	2 (5.88)	1 (1.96)	
Missing data (%)	1 (5.00)	1 (2.94)	3 (5.88)	
Vocational education				
Apprenticeship (%)	9 (45.00)	13 (38.24)	20 (39.22)	0.2138
Technical College (%)	4 (20.00)	3 (8.82)	3 (5.88)	
University of Applied Sciences (%)	3 (15.00)	2 (5.88)	4 (7.84)	
University (%)	0 (0,00)	9 (26.47)	12 (23.53)	
No (%)	0 (0.00)	1 (2.94)	1 (1.96)	
Missing data (%)	4 (20.00)	6 (17.65)	11 (21.57)	
Age: Mean (SD)	59.8 (9.8)	60.3 (9.5)	56.6 (7.9)	0.1460
Years since first diagnosis: Mean (SD)	1.9 (0.9)	2.2 (0.8)	1.9 (0.8)	0.1273
Surgery: yes/ %	20/ 100.0	34/ 100.0	51/ 100.0	
Chemotherapy: yes/ %	12/ 60.00	22/ 64.71	19/ 37.25	0.0501
Years since chemotherapy: Mean (SD)	2.1 (0.7)	1.6 (0.6)	1.8 (0.7)	0.1372
Radiotherapy: yes/ %	14/ 70.00	29/ 85.29	38/ 74.51	0.6000
Antihormonal therapy: yes/ %	17/ 85.00	23/ 67.65	32/ 62.75	0.4947
Mistletoe therapy: yes/ %	6/ 30.00	7/ 20.59	13/ 25.49	0.6797

Numbers and percentages concerning patients which started the intervention (T0)

**Table 3:** Baseline values of Cancer Fatigue Scale (CFS-D) and Pittsburgh Sleep Quality Index (PSQI)

	Aerobic therapy	Multimodal therapy	Combination therapy	*p*-value
CFS-D total score				
Baseline	33.5 (8.8)	33.0 (7.7)	34.3 (8.1)	0.6065
PSQI total score				
Baseline	10.0 (3.5)	10.9 (3.5)	10.3 (3.9)	0.5489

Mean (SD)

AT consisted of trainer-guided treatment of 360 min. Home-based training was on average practiced for 223 min/ week (SD = 179) which corresponds to a low-level adherence exercise rate of 67% (3 × 30 min/ week). The intervention program of the CT group comprised 1810 min. In the home-based exercise training the CT patients practiced on average for 155 min/ week (low-level exercise rate of 52.3%), and home-based eurythmy therapy for ten minutes over 6.4 days/ week. The MT group was invited to participate in a 1450-min program. Here, the minimal home-based eurythmy practice of ten minutes was documented on average in 6.5 days/ week.

**Fig. 3:**
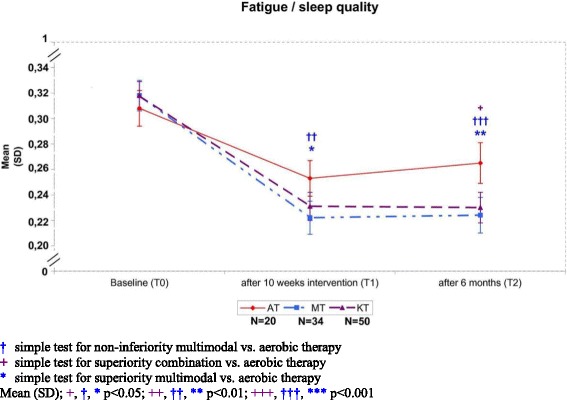
Presentation of the combined outcome (PC-score) of fatigue (CFS-D)/sleep quality (PSQI) at baseline (T0), after 10 weeks intervention (T1) and 6 months later (T2). High values show high fatigue burden and sleep disturbances. The *colored asterisk* indicates significantly reduced fatigue/sleep disturbances. *Red solid line*: AT; *blue* *dashed-dotted* *line*: MT; *purple dashed line*: CT. Higher PC-scores refer to worse complaints

The standardized effect sizes of the primary outcome in the AT, MT and CT group for change from baseline to T1 were 0.48, 1.11, and 0.92; and 0.24, 1.06, and 0.96, for the change from baseline to T2 (Fig. 3), respectively. In the closed testing procedure the global null hypothesis of no overall effect at T1 regarding superiority of CT and non-inferiority of MT versus AT could significantly be rejected (df = 2; F = 4.68; *p* = 0.0115). Similarly, in the subsequent pair-wise comparison MT could be shown to be non-inferior to AT (with regard to the non-inferiority threshold δ) (Δ_PC incl. δ_ = −0.0476, 95%-CI [−0.0812; −0.0140]; *p* = 0.0059). However, superiority of CT over AT could not be demonstrated (Δ_PC_ = −0.0247, 95%-CI [−0.0558; 0.0064]; *p* = 0.1187). Hence, the confirmatory analysis was terminated and all subsequent tests were carried out with an explorative intention. Here, even a significant improvement over AT could be shown for the MT group (Δ_PC_ = −0.0369, 95%-CI [−0.0705; -0.0034]; *p* = 0.0314). At T2, 6 months after the intervention, both MT and CT were significantly superior to AT (CT vs. AT: Δ_PC_ = −0.0436, 95%-CI [−0.0781; −0.0091]; *p* = 0.0137; MT vs. AT: Δ_PC_ = −0.0538, 95%-CI [−0.0910; −0.0166]; *p* = 0.0050) (Fig. 3).

The standardized effect sizes of the CFS-D in the AT, MT and CT group for the change from baseline to T1 were 0.72, 1.16, and 0.98, respectively; and 0.39, 1.18, and 0.90 for T2, respectively. For the PSQI, the corresponding effect sizes values for T1 were 0.29, 0.83, and 0.64, respectively; and 0.09, 0.69, and 0.79 for T2, respectively. In separate analyses of CFS-D and PSQI the MT group at both time points T1 and T2 demonstrated a superior effectiveness over AT (except for CFS-D at T1 where only non-inferiority could be shown), whereas for CT no statistical superiority over AT could be shown with the exception of PSQI at T2 (Table 4). In further differentiated analyses of the questionnaire subscales the MT groups showed the strongest effects in the CFS-D subscales, statistically superior to AT for affective (at T1 and T2) and physical fatigue (T2) and non-inferior for cognitive fatigue (T1, T2). For the CT group strong and significant improvements over AT were observed at T2 for the PSQI subscales ‘subjective sleep quality’, ‘sleep latency’, and ‘sleep duration’. Regarding the PSQI subscales, for MT only for ‘sleep latency’ at T1 and ‘daytime sleepiness’ at T2 significant differences to AT were found. In no parameter and at no time point was AT statistically superior to any of the experimental treatment arms, and only once (PSQI ‘sleep duration’) was the mean AT value marginally better compared to MT and CT (Table 4).

**Table 4:** Differences from baseline values at T1 and T2

	Aerobic therapy	Multimodal therapy	*p*	Combination therapy	*p*
CFS-D total score					
T1.10-T0.01	−6.3 (7.4)	−8.9 (7.1)^†^	0.053	−7.9 (9.0)	0.285
T2-T0.01	−3.4 (9.1)	**−9.1 (7.9)** ^††^	**0.004**	−7.3 (10.2)	0.111
PSQI total score					
T1.10-T0.01	−1.0 (1.8)	**−2.9 (2.9)** ^††^	**0.044**	−2.5 (3.4)	0.077
T2-T0.01	−0.3 (2.8)	**−2.4 (4.0)** ^†^	**0.047**	**−3.1 (3.2)**	**0.007**
CFS-D physical					
T1.10-T0.01	−3.2 (3.8)	−3.7 (3.5)	0.359	−3.2 (4.2)	0.777
T2-T0.01	−0.9 (4.1)	**−4.1 (4.2)** ^††^	**0.003**	−3.1 (4.6)	0.075
CFS-D cognitive					
T1.10-T0.01	−2.0 (2.3)	−3.0 (3.1)^†^	0.062	−2.5 (3.1)	0.556
T2-T0.01	−2.0 (3.6)	−2.9 (3.2)^†^	0.055	−2.6 (3.7)	0.431
CFS-D affective					
T1.10-T0.01	−1.1 (1.9)	**−2.2 (2.0)** ^††^	**0.004**	**−2.2 (2.6)**	**0.018**
T2-T0.01	−0.5 (2.1)	**−2.2 (1.7)** ^†††^	**0.004**	−1.6 (3.4)	0.079
PSQI subjective sleep quality					
T1.10-T0.01	−0.5 (0.5)	−0.6 (0.6)	0.842	−0.5 (0.8)	0.757
T2-T0.01	−0.1 (0.7)	−0.6 (0.8)^†^	0.054	**−0.7 (0.7)**	**0.041**
PSQI sleep latency					
T1.10-T0.01	0.0 (0.8)	**−0.7 (0.9)** ^††^	**0.004**	−0.3 (0.8)	0.066
T2-T0.01	0.1 (0.8)	−0.4 (1.0)	0.155	**−0.4 (1.1)**	**0.0296**
PSQI sleep duration					
T1.10-T0.01	−0.5 (0.7)	−0.2 (0.8)	0.497	−0.2 (1.0)	0.517
T2-T0.01	−0.1 (0.6)	−0.4 (1.2)	0.226	**−0.6 (1.1)**	**0.036**
PSQI sleep efficiency					
T1.10-T0.01	−0.3 (0.8)	−0.7 (1.3)	0.258	−0.6 (1.1)	0.335
T2-T0.01	0.1 (1.0)	−0.3 (1.3)	0.759	−0.7 (1.1)	0.0597
PSQI sleep disturbances					
T1.10-T0.01	−0.1 (0.3)	−0.3 (0.6)	0.411	−0.2 (0.6)	0.463
T2-T0.01	−0.1 (0.6)	−0.3 (0.6)	0.2497	−0.1 (0.7)	0.397
PSQI taking hypnotics					
T1.10-T0.01	0.3 (0.9)	−0.1 (0.5)^†^	0.109	−0.1 (0.6)	0.109
T2-T0.01	−0.1 (0.9)	0.1 (0.6)	0.398	−0.1 (0.6)	0.637
PSQI daytime sleepiness					
T1.10-T0.01	−0.3 (0.8)	−0.5 (0.7)^†^	0.066	−0.5 (0.6)	0.326
T2-T0.01	−0.2 (0.7)	**−0.5 (0.8)** ^††^	**0.024**	−0.5 (0.8)	0.180

*p*-value for test for superiority vs. aerobic therapy

^†^test for non-inferiority vs. aerobic therapy

Mean (SD); ^†^ *p* < 0.05, ^††^ *p* < 0.01; ^†††^ *p* < 0.001

### Safety

Over the course of the entire study period 115 adverse events (AEs) were documented, 87 of which were TESS. Five TESS were classified as adverse therapy reactions with an at least possible causal connection to one of the study interventions: ‘back pain’, reported once in each of the treatment groups; ‘dizziness during eurythmy’; and ‘increasing exhaustion through sleep restriction/stimulus control’, both occurring in the MT group. With regard to individual interventions, all adverse therapy reactions occurred ‘very rarely’ (<0.01%) to ‘rarely’ (0.01–0.1%) except of ‘pain’ in the AT group which occurred ‘occasionally’ (0.1–1%). In relation to total intervention time, AEs not related to study treatments occurred less frequently in the MT group (MT: 0.035/ week, 95%-CI [0.018; 0.067]; AT: 0.054/ week, 95%-CI [0.025; 0.1198]; CT: 0.080/ week, 95%-CI [0.056; 0.113]; compared to CT this difference is significant (*p* = 0.0229). Moreover, 47 and 15% of all TESS in the CT group were of a medium and severe nature, respectively, while these frequencies in the MT group were only 13 and 6%, respectively; in the AT group intermediate frequencies of 27 and 9% were observed, respectively.

## Discussion

This is the first effectiveness study that evaluates the recently developed multimodal concept consisting of psycho- and sleep-education and mindfulness-based eurythmy- and art therapy [20]. In the confirmatory test procedure the multimodal therapy was formally non-inferior and in combination with aerobic training not superior to AT in reducing fatigue/disturbed sleep compared to standard aerobic training after 10 weeks, whereas in our explorative analyses, we found a significant reduction in the MT group at T1 and in both experimental groups 6 months later, with strong effect-sizes both in MT and in CT. Both scales of the PC-score of CFS-D and PSQI contributed in a comparable manner to the therapeutic effects. While in the MT arm improvements were mainly based on the affective and physical subscales of the CFS-D and the daytime sleepiness scale of the PSQI, in the CT arm several subscales of the PSQI (‘subjective sleep quality’, ‘sleep latency’, and ‘sleep duration’) contributed preferentially to the observed improvement at T2.

The presented effects are higher than the minor to moderate effect sizes from standard endurance training, cognitive behavioral therapy [40], and mindfulness-orientated yoga [41] or mindfulness-based stress reduction [42], and point therefore to synergistic sustainable effects on sleep quality and CRF. Corresponding to the increasing evidence of the relationship between CRF and sleep, a pathophysiological model of CRF focuses on the interaction of chronic inflammation, sleep disorder, disrupted rhythm, neuroendocrine disturbances such as cortisol rhythm flattening or reduced responsiveness of cortisol to stress and consecutive central nervous system dysregulations such as elevated corticotropin-releasing hormone, reduced growth factor or 5-Hydroxytryptamin on CRF, cognitive dysfunction, impaired sleep or depression [43]. In breast and lung cancer patients, a polysomnography study reports a reduced sleep-efficiency, more wake-after-sleep-onset compared to healthy control and body movements compared to patients with psycho-physiological insomnia, underlining the non-restorative sleep in these patients [44]. Even if a small study of breast cancer patients undergoing chemotherapy found no other physiological changes in polysomnography apart from an increase in fatigue, prolongation of total sleep time and a persistent unchanged low-level slow wave sleep [45], in different studies subjective and actigraphic sleep-quality decline significantly under chemotherapy [46].

As a conclusion, these aspects underline firstly the complexity of CRF and secondly the weak to moderate effects of actual evidence-based individual treatments, which implicate that a multimodal approach such as implemented as standard therapy in chronic pain management [47] could be an adequate concept. Even though a multimodal mind-body based intervention program consisting of nutrition counseling, relaxation exercises, physical exercises, stress reduction, basics of cognitive restructuring and hydrotherapy did not show superiority in breast cancer survivors with CRF compared to aerobic training [17] we decided to implement our program with a stronger focus on sleep resynchronization and cancer-specific psycho-education [20].

Our hypothesis is that sleep education, restriction, and stimulus control together with the other treatment components improve PSQI-sleep quality in reducing sleep onset latency, sleep duration, and improvement of sleep continuity. Based on the reduced sleep de-synchronization, daytime sleepiness and fatigue can improve. This is consistent with the results of two randomized controlled trials (RCTs) in breast cancer patients with CRF [10, 11] showing clear improvements of sleep quality, but fewer effects on CRF. In our study, however, PSQI-sleep quality and CFS-D fatigue contribute equally to the composite PC-score. This finding points to different effecting mechanisms. Along with the stabilization of the rest/activity rhythm we wanted to strengthen patients’ self-management and self-regulation with the help of psycho-education, and patients’ active stress-management competence with an impact on (affective) CRF [20] with the practice of mindfulness-oriented eurythmy and painting therapy. For mindfulness-based stress reduction and yoga, small effect sizes in CRF have been published [41] with a first indication of concomitant reduction in inflammation-related gene expression [48]. Even though no CRF-related trial has been conducted for eurythmy therapy there are first indications that it can have a stress-reducing [49], and a heart-rate variability- and rhythm stabilizing effect [50]. For painting- or art-therapy a review found positive effects on health-related quality of life, depressive and anxiety symptoms and, in qualitative studies, hints for personal development [51]. Hence, we interpret the strong effect size in the multimodal treatment group as synergistic effects of the different treatment components, although their distinct contribution remains unclear. The reduced and delayed therapy effects in the combined group could be related to the increased frequency and the severity of unspecific side effects. With the combination therapy, the higher risk of these unspecific side effects could be related to endurance training as well as to ‘over-stimulation’. Based on the low-level of TESS all evaluated therapies are well tolerated and can be regarded as very safe. Nevertheless, the intensity of aerobic training should be reduced in the combination therapy. Even if the efficacy of the multimodal approach needs still to be proven in another trial two positive studies strongly support its sustainable effects [20].

Adverse events in general were rarely documented, and only exceptionally regarded as related to any study intervention. However, the relative higher frequency and severity of AEs in the CT arm, statistically detectable when compared to MT, indicates that there might have been an excessive demand regarding the physical capabilities at least in some of these patients. Together with the impression that treatment effects seem in general to be less pronounced in the CT arm compared with MT this raises questions regarding the appropriate amounts of multimodal therapy and aerobic training for optimal CT.

The inclusion criteria strictly concentrated on patients with CRF, but the exclusion of metastasized breast cancer patients in particular may limit the generalization of the results. Our study has some other limitations such as the small sample size and relatively high drop-out rate in the preference aerobic arm. Replacement of so high a percentage of missing data by last-value-carried-forward can introduce a strong bias. Yet, it is our impression that patients did not drop out randomly but due to dissatisfaction or overstraining with regard to the therapies. In this case last-value-carried-forward replacement, which means ‘no change from baseline’, is a justifiable approach as could be confirmed in an inspection of individual patient measurements. Another concern is that the patients in the aerobic standard therapy group received a shorter, trainer-guided treatment of 360 min compared to the combined- and multimodal treatment arms. This could be understood and discussed in the sense of receiving less attention. Nevertheless, the therapeutic effect sizes both at T1 and T2 are beyond or in the range of other published meta-analyses in breast cancer survivors [9, 52, 53]. Although the overall drop-out rate of 20% is high, it is within the range of other endurance training studies [54]. The four-fold respective five-fold longer intervention time of the multimodal- and combined treatment are related to corresponding higher costs of therapists. Finally, the principal distinction between patients actively pursuing a certain therapy and patients passively randomized into this therapy could introduce not only bias but, at worst, a negative interaction between allocation regime and treatment. No such interaction was observed, yet a detailed examination and publication of these conditions is in preparation.

## Conclusions

The presented study indicates non-inferiority and, based on exploratory results, superiority of a multimodal approach in the treatment of chronic cancer-related fatigue compared to the standard aerobic therapy. This suggests that a multimodal therapy concept consisting of movement therapy, behavior- and mindfulness-based therapies might have the potential to become standard treatment. Therefore a further confirmative RCT is indicated which should investigate the long-term outcome alongside cost-effectiveness.

## Abbreviation

AEs: Adverse events
AT: Aerobic training
BC: Breast cancer survivors
CFS: Cancer Fatigue Scale
CFS-D: Cancer Fatigue Scale (German version)
COPD: Chronic obstructive pulmonary disease
CRF: Cancer-related Fatigue
CT: Combination therapy
HADS: Hospital Anxiety and Depression Scale
ITT: Intention-to-Treat analysis
LOCF: Last-value-carried-forward
MT: Multimodal therapy
NYHA: New York heart association functional classification
PC-score: Principal component score
PS: Propensity scores
PSQI: Pittsburgh sleep quality index
RCT: Randomized controlled trial
SAE: Serious adverse event
SD: Standard deviation
SE: Sleep education/restriction
T0: Time baseline measurement
T1: Time after 10 weeks intervention
T2: Time after 6 months intervention
TESS: Treatment-emergent signs and symptoms
TSH: Thyroid-stimulating hormone
