# Correction to: Impact of a combined multimodal-aerobic and multimodal intervention compared to standard aerobic treatment in breast cancer survivors with chronic cancer-related fatigue - results of a three-armed pragmatic trial in a comprehensive cohort design

**DOI:** 10.1186/s12885-020-07679-3

**Published:** 2020-12-01

**Authors:** Matthias Kröz, Marcus Reif, Augustina Glinz, Bettina Berger, Andreas Nikolaou, Roland Zerm, Benno Brinkhaus, Matthias Girke, Arndt Büssing, Christoph Gutenbrunner, On behalf of the CRF-2 study group

**Affiliations:** 1 Department of Internal Medicine, Havelhöhe Hospital, Kladower Damm 221, D-14089 Berlin, Germany; 2 Research Institute Havelhöhe, Kladower Damm 221, D-14089 Berlin, Germany; 3 Institute for Social Medicine, Epidemiology and Health Economics, Charité Universitätsmedizin Berlin, Charité CCM, 10098 Berlin, Berlin Germany; 4 nstitute for Integrative Medicine, University of Witten/Herdecke, Gerhard-Kienle-Weg, 58313 Herdecke, Germany; 5 Society for Clinical Research, Hardenbergstraße 20, 10623 Berlin, Germany; 6 Clinic for Rehabilitative Medicine, Hannover Medical School, Carl-Neuberg-Straße 1, 30625 Hannover, Germany

**Correction to: BMC Cancer 17, 166 (2017)**

**https://doi.org/10.1186/s12885-017-3142-7**

Following publication of the original article [1], the authors reported the following errors:

1) In Fig. 3 the y-axis is incorrectly labeled as ‘Mean (SD)’ instead of ‘Mean (standard error)’, since Fig. 3 in fact shows estimates of mean and standard error of the mean. Figure 3 has been corrected in this correction article. The authors have taken the opportunity to include an editing change by denoting the combination therapy arm as ‘CT’ instead of ‘KT’.

**Fig. 3:**
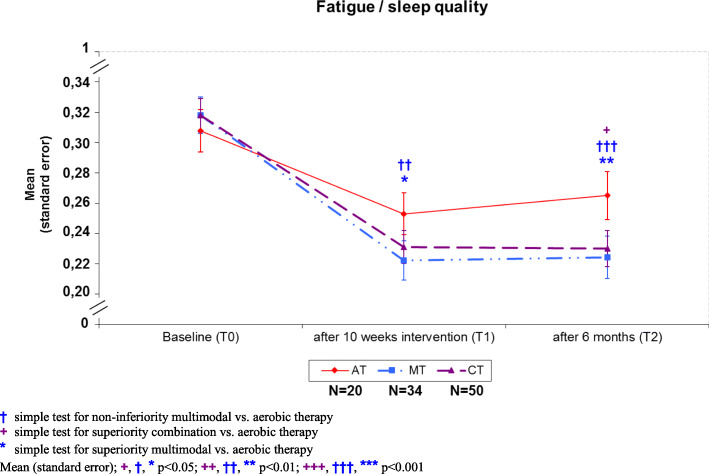
Presentation of the combined outcome (PC-score) of fatigue (CFS-D)/sleep quality (PSQI) at baseline (T0), after 10 weeks intervention (T1) and 6 months later (T2). High values show high fatigue burden and sleep disturbances. The *colored asterisk* indicates significantly reduced fatigue/sleep disturbances. *Red solid line*: AT; *blue dashed-dotted line*: MT; *purple dashed line*: CT. Higher PC-scores refer to worse complaints

2) There are some discrepancies in the first paragraph of the ‘Study-group characteristics’ section caused by a mix-up of ITT analyses with analyses based on the full set of initially included patients. In fact, the mean age values range from 56.6 to 60.3 instead of 56.4 to 58.8 years (as correctly shown in Table 2); and the *p*-values indicating significant group differences of height, rehabilitation, and other disorders are 0.0325, 0.0312, and 0.0369 instead of 0.0168, 0.027, and 0.0313, respectively. None of these changes impair the study outcome or its interpretation in any way.
